# Editorial: Real-world data and real-world evidence in hematologic malignancies

**DOI:** 10.3389/fonc.2023.1232980

**Published:** 2023-06-09

**Authors:** Michele Malagola, Robert Ohgami, Raffaella Greco

**Affiliations:** ^1^ Unit of Blood Diseases and Stem Cell Transplantation, Azienda Socio Sanitaria Territoriale (ASST)-Spedali Civili of Brescia, Department of Clinical and Experimental Sciences, University of Brescia, Brescia, Italy; ^2^ The University of Utah, Salt Lake City, UT, United States; ^3^ Unit of Hematology and Bone Marrow Transplantation, Istituto di Ricovero e Cura a Carattere Scientifico (IRCCS) San Raffaele Scientific Institute, Milano, Italy

**Keywords:** real-world data, real-world evidence, hematological malignancies, clinical trials, real-life

Advances in the availability and analysis of real-world data (RWD) have substantially contributed to generate robust real-world evidence (RWE), thus supporting the development of recommendations/guidelines and regulatory decisions closer to real-life experience.

RWD and RWE are closely related but not interchangeable. RWD are data related to patient health status and/or the delivery of health care routinely collected from a variety of sources, processed and analyzed through advanced analytical methods such as data mining, machine learning, and artificial intelligence (1). RWE refers to the meaningful insights and conclusions extracted from RWD (2).

In 2023, clinical care guidelines and available treatments are changing so rapidly that making decisions based only on clinical trial data is becoming outdated in many areas, including hematology. Moreover, in the transplant and cellular therapy settings, clinical patient care is generally localized, practices may differ across countries and centers, generating interest towards harmonization (3). RWD is paving the way towards generating insights that can drive decisions in life sciences and healthcare research. Indeed, RWD can help validate findings from clinical trials, evaluating the effectiveness and safety of treatments, strategies, and programs in a real-world setting. This may be useful to identify patient subgroups that may benefit more from specific treatments. RWD can also support research and development, including the design of clinical trials and the identification of unmet healthcare needs. In this context, RWE generated from RWD may facilitate appropriate clinical decisions, recommendations, and healthcare planning. These outcomes can then be used for a variety of decisions and drive improvements in patient care, including more accurate diagnoses, better algorithms, and personalized treatments.

Indeed, RWD are expected to play an increasingly important role in healthcare research and decision-making in the years to come, as witnessed by this Frontiers in Immunology Research Topic on “*RWD and RWE in hematological malignancies*”.

Twenty articles have been accepted and included in this Research Topic. Twelve are published in the form of Original Research Articles, 5 are Case Reports, 2 Brief Research Reports and 1 is a Systematic Review.

Among the Research Articles, 6 focus on Acute Leukemias, 3 on Lymphomas, and 3 on Multiple Myeloma. Four out of the 6 articles on Leukemias are focused mainly on the biology of the disease: this is a clear evidence that the biology study of these diseases is still of major interest in the community of Hematologists. In particular, these studies focus on relatively rare entities and/or peculiar biological findings: early T-precursor Acute Lymphoblastic Leukemias (ETP-ALL; Chen et al.), Myeloid Sarcoma (MS; Xing et al.), bone marrow fibrosis in acute myeloid leukemia (AML; Zhang et al.), and lipid profile in AML (Bai et al.). In particular, these RWD highlight some crucial aspects: the prognosis of ETP-ALL following allogeneic stem cell transplantation seems similar to that of non-ETP ALL (Chen et al.), the clinical features and the prognosis of patients with MS involving hematopoietic vs non-hematopoietic sites is different (Xing et al.), bone marrow fibrosis is an independent adverse prognostic factor in AML patients (Zhang et al.), and lipid profile together with clinical characteristics of AML patients may improve patients’ prognostication (Bai et al.). Although these findings should be confirmed in larger and possibly prospective studies, they represent interesting aspects to be considered in the clinical management of our patients. Two other Research Papers on AML are clinically oriented and covers peculiar aspects. Zhang et al. explore the effects of intensive chemotherapy on megakaryoblast AML nonrelated to Down’s syndrome: although the prognosis of this peculiar entity remains poor, intensive chemotherapy may have some advantages in terms of long term survival. The other research article on AML focuses on a classical dilemma in the field of AML: is standard dose cytarabine-based consolidation chemotherapy superior to high dose? To address this issue, Wang et al. analyze a series of 183 patients younger than 60 years, suggesting that consolidation with high-dose cytarabine leads to superior outcomes, particularly in intermediate-risk group according to the 2022 ELN classification.

Moving to the three Research Articles on Lymphomas, it is notable that they are all dedicated to Central Nervous System Lymphomas (CNSL), suggesting that this group of lymphomas represents a clear hot topic of research and study. Wu et al. propose a prognostic scoring model, including lesion number, beta-2 microglobulin, systemic inflammation response index and Karnofsky performance status. This score has been tested on a cohort of 122 patients with PCNSL, 72 of whom were used to develop the model and 50 of whom were used as a validation set. Three groups of patients with different long-term outcome are identified, and this is reproducible across different treatments (chemotherapy vs Bruton’s tyrosine kinase inhibitors) and in elderly patients. The topic of the best treatment for PCNSL (chemotherapy vs. radiotherapy) is covered in the manuscript by Yang et al., in which 105 relapsed/refractory PCNSL are addressed to salvage treatment with chemo or radiotherapy. Interestingly, the overall response rate is higher in patients treated with radiotherapy both in the relapsed and in the refractory group. Moreover, age, cerebral spinal fluid protein level and ocular involvement are factors associated with impaired outcome. Overall, these data clearly suggest that the prognosis of PCNSL is influenced by several different biological and clinical factors and that conventional therapy (chemo and radiotherapy) still play a major role in the management of the advanced phase of the disease. The topic of central nervous system (CNS) involvement has been explored also in the manuscript by Jeong et al. In particular they focus on CNS localization in diffuse large B cell lymphomas and explore the feasibility and efficacy of autologous stem cell transplantation (ASCT) following high-dose methotrexate reinduction. This treatment algorithm was safely performed on 43 patients. After ASCT, 17 patients (39%) maintain the complete remission (median follow up 14.7 months) suggesting that this treatment option is feasible. This result is of interest, because the salvage treatment of patients with CNS involvement is an unmet clinical need, as CAR-T cell therapy, at present, is still a matter of debate.

The three Research articles on Multiple Myeloma covers distinct areas. The adverse prognostic impact of chromosome 1q21 gain in patients treated with bortezomib-based therapy is underlined in the manuscript by Liu et al. Xu et al. cover a very important aspect of multiple myeloma treatment in the era of new molecular target drugs: the socioeconomic status strongly influences survival disparities, suggesting that non-Hispanic, white, married, insured and urban patients have an increasing linear trend in survival benefits. Finally, Bao et al. suggest the usefulness of a machine learning tool to predict survival in elderly patients with multiple myeloma without genomic data and showed that patients who received an immunomodulator agent as maintenance had the best survival.

The two brief research reports are highly interesting. Morin et al. cover the topic of post-allogeneic stem cell maintenance (allo-SCT) with Sorafenib in FLT3-ITD positive AML. 30 patients receive post-transplant maintenance and data on long-term survival are intriguing: after 12 months of median follow up, median overall survival is not reached. The topic of post-transplant maintenance is of high interest now that we have molecular target drugs, such as FLT3 inhibitors, but also azacitidine and venetoclax. It is highly probable that the scenario of the next future will change, and that the great majority of AML patients will receive an individualized maintenance following allo-SCT. The other brief Research Report covers the topic of defibrotide prophylaxis of sinusoidal obstruction syndrome (SOS) in adults submitted to allo-SCT following Inotuzumab Ozogamicin-based treatment (Giglio et al.). Seven patients were treated, four of whom received a double-alkylator based conditioning regimen. Three patients developed fatal SOS and all the three patients received the double-alkylator conditioning regimen. Several data suggest that defibrotide plays a crucial role in the treatment of SOS, but further data are warranted to better define its role in prophylaxis.

This Research Topic also includes an interesting review, focusing on agents contributing to secondary immunodeficiency development in patients with chronic lymphoproliferative disorders (Jolles et al.). As expected, multiple myeloma patients treated with monoclonal antibodies, as well as patients with chronic lymphocytic leukemia and non-Hodgkin’s lymphomas treated with a tyrosine kinase inhibitors are those at major risk of developing infectious complications. Moreover, the Authors reported a global under-reporting of hypogammaglobulinemia and lymphocytopenia before and during therapies: this suggests that a higher attention should be addressed to this aspect, as infectious complications represent a major cause of morbidity and mortality, as well as healthcare costs.

Finally, 5 case reports are included in the Research Topic (Giglio et al., Pederzolli et al., Wang et al., Wang et al., and Ji et al.). Without going into details (please refers to the electronic links), all these reports cover interesting and peculiar clinical situations: ponatinib as bridge to CAR-T (Giglio et al.), intravitreal methotrexate in ocular acute lymphoblastic leukemia (Pederzolli et al.), Zanubrutinib-induced dermatological toxicity (Wang et al.), association of acute promyelocytic leukemia and metachronous multiple primary carcinoma (Wang et al.) and Langerhans cell histiocytosis of the thymus and heart (Ji et al.). Although rare entities, these case reports represent valid tools for clinicians who may be involved in the management of patients with similar conditions.

In conclusion, we think that this Research Topic helped us to collect several interesting articles on real-life studies, covering different aspects of different hematological diseases. Taken together these data suggest that real life is still an important way to collect informative data useful for the design of prospective, controlled trials, that are fundamental to confirm any preliminary result.

## Author contributions

All authors listed have made a substantial, direct, and intellectual contribution to the work and approved it for publication.
